# Prevalence of metabolic syndrome among breast cancer survivors in East Coast of Peninsular Malaysia

**DOI:** 10.1186/s12889-021-10288-9

**Published:** 2021-01-28

**Authors:** Mohd Razif Shahril, Syed Amirfaiz, Pei Lin Lua, Ali Nurnazahiah, Nor Syamimi Zakarai, Ving Lok Kow, Aryati Ahmad, Suhaina Sulaiman

**Affiliations:** 1 Centre for Healthy Ageing and Wellness (H-CARE), Faculty of Health Sciences, Universiti Kebangsaan Malaysia, Jalan Raja Muda Abdul Aziz, 50300 Kuala Lumpur, Malaysia; 2 Faculty of Health Sciences, Universiti Sultan Zainal Abidin, Gong Badak Campus, Kuala Nerus, 21300 Kuala Terengganu, Terengganu Malaysia; 3 Faculty of Pharmacy, Universiti Sultan Zainal Abidin, Besut Campus, Besut, 22200 Kuala Terengganu, Terengganu Malaysia

**Keywords:** Metabolic syndrome, Triglycerides, Glucose, HDL-c, Breast cancer, Survivors

## Abstract

**Background:**

To date, limited data are available on metabolic syndrome prevalence among breast cancer survivors in Malaysia. Therefore, this study was conducted to determine the prevalence of metabolic syndrome and abnormal metabolic syndrome components among breast cancer survivors in East Coast of Peninsular Malaysia.

**Methods:**

This cross-sectional study included 95 breast cancer survivors (age 53.7 ± 7.6 years) who have completed main cancer treatments for ≥6 months. Cancer survivors were recruited from two main government hospitals in Kelantan and Terengganu using a purposive sampling method.

**Results:**

According to the Harmonized criteria, the metabolic syndrome prevalence was 50.5%. Among those with metabolic syndrome, the most prevalent abnormal metabolic components were triglycerides (91.2%), fasting blood glucose (79.6%) and HDL-c level (78.4%). Except for total cholesterol and LDL-c, all other metabolic syndrome components were significantly different (*p* < 0.05) between those with and without metabolic syndrome. Significant differences between metabolic syndrome and non-metabolic syndrome groups were found for weight, BMI, waist circumference, body fat percentage and cancer stages (*p* < 0.05). However, no significant relationship was reported between sociodemographic, clinical parameters and metabolic syndrome among breast cancer survivors in this study.

**Conclusions:**

Metabolic syndrome was highly prevalent among breast cancer survivors. It is recommended for health care professionals to closely monitor and improve the triglycerides, blood glucose and HDL-c level of the breast cancer survivors under their care to control the detrimental effect of metabolic syndrome.

**Supplementary Information:**

The online version contains supplementary material available at 10.1186/s12889-021-10288-9.

## Background

The growing number of data have shown that metabolic syndrome (MetS) and its independent components are related with plethora of cancers, including a higher risk of having breast cancer [1–3]. Similarly, breast cancer survivors were also reported to be susceptible to MetS [4, 5]. In Malaysia, the prevalence of MetS among breast cancer patients was reported at 37.8% [6]. In other Asian and Western countries, MetS prevalence among breast cancer survivors were reported at comparable magnitude in countries such as India (31.1 to 40.0%) [7, 8], China (32.9%) [9], Korea (43.9%) [10], USA (26.1%) [11], and Brazil 48.1% [12]. Prevalence of MetS among breast cancer patients in Denmark was rather lower (15.1%) than other reported studies [13].

In contrast, there are a lot more studies conducted on MetS prevalence among the general population. In Malaysia, the prevalence of MetS has been extensively reported [14]. To summarize, MetS prevalence among general Malaysian women in three nationwide studies were reported to range between 30.1 to 43.7%% [15–17]. Higher risk MetS was also reported to be linked with higher age, being obese, Indian ethnicity, lower education level, unemployment and shift workers [14]. Meanwhile, MetS prevalence among general women population in other Asian and Western countries were at similar rate such as India (43.2%) [18], China (34.2%) [19], Thailand (36.4%) [20], Spain (30.7%) [21], Norway (34.2%) [22] and Netherland (44.0%) [23]. Uniquely, Korea only reported 11.4% of MetS prevalence among their population [24].

Due to the inter-relationship between MetS and breast cancer, the study on MetS among breast cancer survivors could be a two-pronged investigation to counter these health issues at the same time. Nevertheless, up until today, limited data on the prevalence of MetS among breast cancer survivors in Malaysia have been published, especially in the East Coast of Peninsular Malaysia. Therefore, this study was conducted to determine the prevalence of MetS and abnormal MetS components among breast cancer survivors in East Coast of Peninsular Malaysia. In view of findings from a systematic review which indicates that breast cancer survivors are more susceptible to MetS [4], it is hypothesized that the prevalence would be much higher compared to healthy population.

## Methods

### Study design and participants

In this cross-sectional study, a total of 95 breast cancer survivors were recruited by using purposive sampling method from the surgical outpatient clinics of two main government hospitals in East Coast of Peninsular Malaysia with highest number of breast cancer cases; Hospital Sultanah Nur Zahirah in Terengganu and Hospital Raja Perempuan Zainab II in Kelantan. The surgical outpatient clinic attends all types of surgical patients and all breast cancer survivors were purposively sampled based on clinic contact list for breast cancer patients. Sample size were calculated using G*Power software version 3.1 with an effect size *g* of 0.144, constant proportion of 0.182 based on a similar study from China [9], considering 95% significance level, 80% power and a 10% margin for incomplete data. The inclusion criteria for breast cancer survivors’ recruitment in this study were; a) Malaysian women; b) adults (≥18 years old); c) have completed the active cancer treatments (surgery, chemotherapy and/or radiotherapy); d) completed at least four rounds of chemotherapy; e) at least 6 months of post-active treatments, and f) able to read and communicate in English or Malay. Breast cancer survivors were excluded if they had secondary, recurrent or stage four breast cancer, were pregnant, or if they had cardiovascular, orthopedic or medical conditions which could be worsened by exercise. Ethical approval was obtained from the Ministry of Health, Malaysia (NMRR-14-1618-23,717-IRR). All potential research participants were briefed on the procedure, risks and benefits of the study. They were also informed that they could decide to drop out at any time of the study. Before data collection could be commenced, verbal and written consent from the breast cancer survivors were obtained.

### Recruitment of breast cancer survivors

After obtaining ethical approval from the Ministry of Health and the administration of both hospitals, the name list of breast cancer survivors was obtained together with their contact numbers from the clinic. All breast cancer survivors were personally contacted to briefly explain the research and queried for inclusion and exclusion criteria. At the same time, all eligible breast cancer survivors were invited to join the study. Those who gave verbal consent were set up for an appointment. During the meetup session, study information sheets and further elaboration on the study procedure were given to all participants before written consent was obtained from each of them. All data were collected between November 2015 to February 2016.

### Metabolic syndrome definitions and measurements

In this study, prevalence of MetS was first investigated by using the World Health Organization (WHO) [25], National Cholesterol Education Program Adult Treatment Panel (NCEP ATP-III) [26], International Diabetes Federation (IDF) [27] and Harmonized diagnostic definitions [28]. However, only the Harmonized definition was used for further analysis and reports regarding MetS prevalence. As suggested by the Harmonized criteria, MetS was diagnosed among breast cancer survivors with at least three out of five metabolic abnormalities. Additionally, breast cancer survivors who have been previously diagnosed with type II diabetes mellitus, or those who were on lipid and antihypertensive medication were also considered in these metabolic abnormalities. Anthropometric measurements were conducted with subjects in light clothing. Waist circumference, height and weight were assessed according to the WHO protocol [29]. Briefly, waist circumference was measured to the nearest 0.1 cm at the iliac crest by using SECA 201 measuring tape (SECA GmbH & Co. KG, Hamburg, Germany). Height measurement of the breast cancer survivors was taken to the nearest 0.5 cm by using SECA 217 mobile stadiometer (SECA GmbH & Co. KG, Hamburg, Germany) while they were standing straight with heels together, arms to the side and head in the Frankfurt horizontal plane [30].

Weight and body fat percentage were measured to the nearest one decimal place using TANITA breast cancer-543 body composition monitor (TANITA Corporation, Tokyo, Japan) while the subjects were standing still with weight equally distributed on both feet. To obtain the blood pressure data, OMRON HEM-7203 electronic blood pressure monitor (OMRON Corporation, Kyoto, Japan) was used. All subjects were in a seated position and the measurements were taken after a 5-min rest. All anthropometric and blood pressure measurements were repeated two times and the average measurements were recorded. The body weight and height data were used to calculate and categorized the body mass index (BMI) (kg/m^2^) of the subjects according to the WHO classification [29].

Fasting blood sampling via venipuncture was scheduled by appointment with patients who fasted at least 8 h. A total of 5 ml blood was drawn by clinic nurse upon consent by patients. Laboratory analyses of the blood samples were carried out to obtain data on levels of triglycerides, high-density lipoprotein cholesterol (HDL-c) and fasting blood glucose. Meanwhile, low-density lipoprotein cholesterol (LDL-c) level was calculated using the Friedewald formula. The fasting blood glucose and lipid profiles analyses were conducted by using a fully-automated chemistry analyzer Olympus AU 400 (Olympus Corporation, Tokyo, Japan) with standard enzymatic and colorimetric methods. Information on sociodemographic profiles of the breast cancer survivors was acquired by using a self-administered questionnaire, whereas additional clinical and treatment data were obtained from the patients’ medical records using data collection form. Both the questionnaire and data collection form were pre-tested prior to actual data collection. The pre-test of questionnaire with patients suggested some amendments to the phrases used to increase clarity and reduce recall bias. Meanwhile data collected from patients’ medical reports by two researchers (AN and NSZ) using the data collection form was found to be consistent with 100% agreement when cross checked by the clinician.

### Statistical analyses

Descriptive statistics were used to summarize demographic, anthropometric, biochemical and clinical data of the study sample. Parameters with normal data distribution were reported as mean with standard deviation, while others were reported as the median and interquartile range (IQR). To compare the differences in clinical, metabolic, sociodemographic and anthropometric characteristics according to MetS status, statistical analyses to compare two independent groups were used namely Chi-square test for categorical data and independent t-test for continuous data. Statistical significance was taken as a *p*-value of less than 0.05. The relationship between characteristics of study sample and metabolic syndrome was also tested using multiple logistics regression with metabolic syndrome status as a dependent variable (outcome) and sociodemographic and clinical variables as covariates. All statistical analysis was conducted by using IBM SPSS for Windows software, version 22.0 (IBM Corp, Armonk, NY, USA). There were no missing data in this study for all variables.

## Results

### Characteristics of breast cancer survivors

A total of 545 breast cancer survivors were listed at the hospital, but majority of them were unable to be reached by phone (44.4%), did not meet study criteria (11.4%), died (8.1%), or refused to participate (6.7%). Of the balance 160 eligible survivors, only 95 were included in the final analysis as 32 could not make it to the hospital during study period and 33 provided incomplete data. Table 1 shows the characteristics of all breast cancer survivors that were included in this study (*n* = 95). Overall, the mean age ± SD of the subjects was 53.7 ± 7.6 years. Most of the cancer survivors were Malay (92.6%), married (72.9%), housewives (34.7%), had a maximum education level of secondary schools (64.2%) and a monthly income of less than MYR 1000 (USD 242) (36.8%). Next, majority of the cancer survivors were postmenopausal (87.4%), had prior experience of breastfeeding (88.4%), did not undergo hormone replacement therapy (83.2%) and had no family history of breast cancer (72.6%). Additionally, more than half of the breast cancer survivors did not take any oral contraceptive pill (54.7%). As there was no smokers or alcohol-drinkers among the breast cancer survivors, the link of these lifestyle factors with the presence of MetS could not be investigated.

**Table 1:** Characteristics of breast cancer survivors included in this study

Characteristics	Total (*n* = 95)	MetS (*n* = 48)	Non-MetS (*N* = 47)	*p*
Sociodemographic profiles				
Age ^a^	53.7 (7.6)	55.1 (7.9)	52.4 (7.2)	0.082
Ethnic				
Malay	88 (92.6)	45 (93.8)	43 (91.5)	0.714
Chinese	7 (7.4)	3 (6.3)	4 (8.5)	
Marital status				
Single	3 (3.2)	0 (0)	3 (6.4)	0.262
Married	74 (77.9)	37 (77.1)	37 (78.7)	
Divorced	2 (2.1)	1 (2.1)	1 (2.1)	
Others	16 (16.8)	10 (20.8)	6 (12.8)	
Education				
Primary	6 (6.3)	2 (4.2)	4 (8.5)	0.214
Secondary	61 (64.2)	34 (70.8)	27 (57.4)	
Tertiary	28 (29.5)	12 (25.0)	16 (34.0)	
Occupation				
Professional	22 (23.2)	8 (16.7)	14 (29.8)	0.106
Support staff	10 (10.5)	6 (12.5)	4 (8.5)	
Self-employed	17 (17.9)	13 (27.1)	4 (8.5)	
Housewife	33 (34.7)	14 (29.2)	19 (40.4)	
Pensioner	13 (13.7)	7 (14.6)	6 (12.8)	
Household income ^b^				
< MYR 1000	35 (36.8)	20 (41.7)	15 (31.9)	0.450
MYR 1000–3000	31 (32.6)	16 (33.3)	15 (31.9)	
> MYR 3000	29 (30.5)	12 (25.0)	17 (36.2)	
Menopausal status				
Premenopausal	12 (12.6)	5 (10.4)	7 (14.9)	0.511
Postmenopausal	83 (87.4)	43 (89.6)	40 (85.1)	
Breastfeeding				
Yes	84 (88.4)	44 (91.7)	40 (85.1)	0.318
No	11 (11.6)	4 (8.3)	7 (14.9)	
Oral contraceptive pills				
Yes	43 (45.3)	19 (39.6)	24 (51.1)	0.261
No	52 (54.7)	29 (60.4)	23 (48.9)	
Hormone replacement therapy				
Yes	16 (16.8)	6 (12.5)	10 (21.3)	0.253
No	79 (83.2)	42 (87.5)	37 (78.7)	
Family history of breast cancer				
Yes	26 (27.4)	14 (29.2)	12 (25.5)	0.691
No	69 (72.6)	34 (70.8)	35 (74.5)	
Clinical and metabolic profiles				
Cancer stages				
I	15 (15.8)	7 (14.6)	8 (17.0)	0.019*
II	55 (57.9)	34 (70.8)	21 (44.7)	
III	25 (26.3)	7 (14.6)	18 (38.3)	
Cancer duration (years) ^a^	6.65 (4.19)	7.23 (5.22)	6.06 (2.71)	0.177
Treatments				
Surgery ^c^	94 (98.9)	48 (100)	46 (97.9)	0.495
Chemotherapy	95 (100)	48 (100)	47 (100)	–
Radiotherapy	86 (90.5)	41 (85.4)	45 (95.7)	0.086
Comorbidities ^e^				
Diabetes	17 (17.9)	13 (27.1)	4 (8.5)	0.018*
Hypertension	21 (22.1)	15 (31.3)	6 (12.8)	0.030*
Heart disease ^c^	2 (2.1)	1 (2.1)	1 (2.1)	1.000
Metabolic profiles ^d^				
Total cholesterol (mmol/L)	6.1 (1.6)	6.0 (1.4)	6.2 (1.6)	0.250
LDL-c (mmol/L)	3.8 (1.5)	3.7 (1.3)	4.0 (1.6)	0.195
HDL-c (mmol/L)	1.4 (0.4)	1.3 (0.4)	1.5 (0.5)	< 0.001*
TG (mmol/L)	1.5 (0.8)	1.8 (0.9)	1.3 (0.6)	< 0.001*
FBG (mmol/L)	5.7 (1.9)	6.5 (3.0)	5.0 (0.9)	< 0.001*
Systolic BP (mmHg)	128 (19)	134 (25)	124 (12)	0.006*
Diastolic BP (mmHg)	79 (13)	80 (13)	78 (11)	0.020*
Anthropometric profiles				
Weight (kg) ^a^	66.0 (12.4)	68.7 (10.0)	63.3 (14.0)	0.032*
Height (cm) ^a^	153.9 (6.0)	154.1 (5.1)	153.6 (6.9)	0.711
Waist circumference (cm) ^a^	88.8 (11.7)	92.2 (9.9)	85.3 (12.4)	0.003*
Body fat percentage (%) ^e^	39.0 (6.9)	40.5 (7.7)	38.2 (8.0)	0.020*
BMI (kg/m^2^) ^a^	27.9 (4.9)	29.0 (4.3)	26.7 (5.2)	0.023*
BMI classification (kg/m^2^) ^c^				
Underweight	3 (3.2)	0 (0)	3 (6.4)	0.138
Normal	20 (21.1)	8 (16.7)	12 (25.5)	
Overweight	43 (45.3)	22 (45.8)	21 (44.7)	
Obese	29 (30.5)	18 (37.5)	11 (23.4)	

Data in number (%), *p-*value derived using Chi-square test

^a^Data in mean (SD), *p-*value derived using Independent t-test

^b^MYR, Malaysian Ringgit; MYR 1000 is equal to USD 242

^c^Data in number (%), *p-*value derived using Fisher’s Exact test

^d^Data in median (IQR), *p-*value derived using Mann-Whitney test

^e^The number of subjects without these comorbidities and complications were not reported in this table

^*^p < 0.05, significantly different

*BMI* Body mass index, *BP* Blood pressure, *FBG* Fasting blood glucose, *HDL-c* High density lipoprotein cholesterol, *LDL-c* Low density lipoprotein cholesterol, *MetS* Metabolic syndrome, *TG* Triglyceride

The majority of the breast cancer survivors had stage II breast cancer (57.9%) and prolonged cancer survival duration, with the mean ± SD of 6.65 ± 4.19 years. Besides chemotherapy, most of them also had undergone surgery (98.9%) and radiotherapy (90.5%). As there was only a portion of breast cancer survivors reported to also be diagnosed with diabetes (17.9%), the median of fasting blood glucose level of all breast cancer survivors was reported to be slightly exceeding the normal value of 5.5 mmol per litre. Following the WHO classification for BMI, majority of the breast cancer survivors were overweight (45.3%), followed by obese (30.5%), normal (21.1%), and underweight (3.2%). Moreover, the high mean of waist circumference (88.8 cm) and the median body fat percentage (39.0%) indicated that the majority of breast cancer survivors tend to have central obesity.

### Prevalence of metabolic syndrome and abnormal metabolic syndrome components

**Fig. 1:**
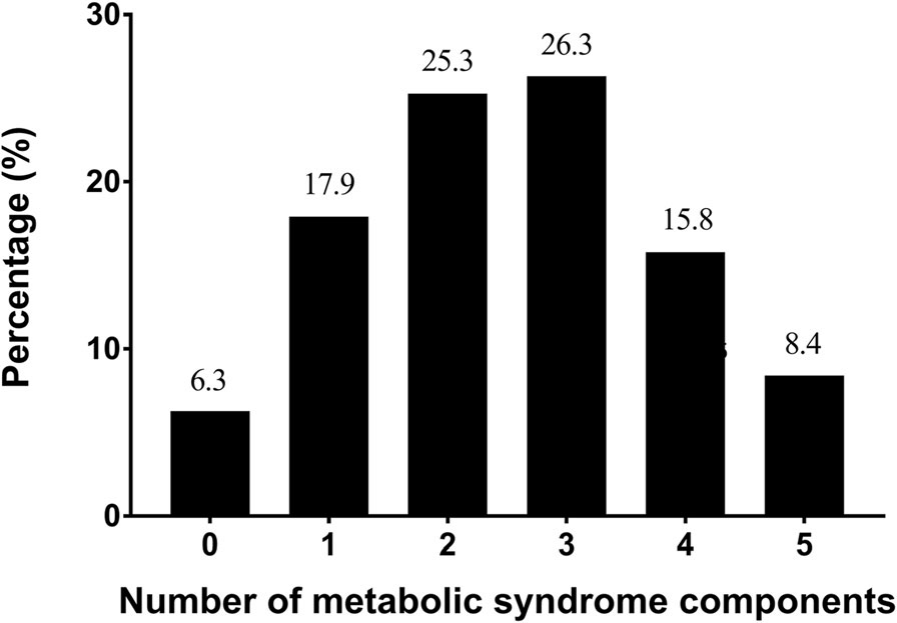
Metabolic syndrome component according to Harmonized criteria

**Fig. 2:**
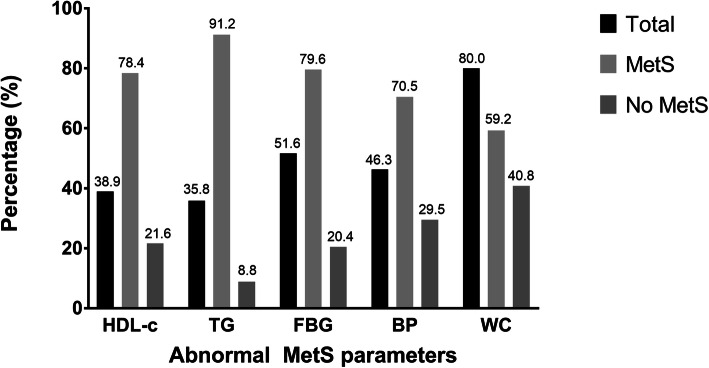
Abnormal metabolic syndrome parameters

The overall prevalence of MetS among breast cancer survivors according to the Harmonized 2009, IDF 2005, ATP III 2001 and WHO 1998 criteria were reported to be 50.5, 48.4, 40.0 and 18.9% respectively (Table 2). When only the Harmonized criteria were considered, around half of the breast cancer survivor population in this study had two (25.3%) or three (26.3%) MetS components (Fig. 1). Meanwhile, Fig. 2 shows the number and percentage of subjects with abnormal MetS parameters in this study. Among all breast cancer survivors, the top three most prevalent abnormal MetS components were waist circumference (80.0%), fasting blood glucose (51.6%) and blood pressure (46.3%), whereas breast cancer survivors with MetS had the highest tendency to have abnormal triglyceride level (91.2%), fasting blood glucose (79.6%) and HDL-c (78.4%).

**Table 2:** Prevalence of metabolic syndrome according to different diagnostic definitions

MetS diagnostic definition	All (*n* = 95) n (%)	95% Confidence Interval
Harmonized (2009)	48 (50.5)	40.6–60.3
IDF (2005)	46 (48.4)	38.6–58.3
NCEP ATP III (2001)	38 (40.0)	30.7–50.0
WHO (1999)	18 (18.9)	12.3–27.9

*IDF* International Diabetes Federation, *MetS* Metabolic syndrome, *NCEP ATP III* National Cholesterol Education Program Adult Treatment Panel III, *WHO* World Health Organization

### Characteristics of breast cancer survivors according to metabolic syndrome status

Analysis of the characteristics of all research participants showed no significant difference in all reported sociodemographic and clinical profiles between those with and without MetS (Table 1). Meanwhile, breast cancer survivors with MetS had significantly higher levels of triglyceride (*p* < 0.001), fasting blood glucose (*p* < 0.001), systolic blood pressure (*p* = 0.006) and diastolic blood pressure (*p* = 0.020), as well as a significantly lower level of HDL-c (*p* < 0.001). In contrast, the total cholesterol and LDL-c levels were not significantly different among those with and without MetS. Significant difference between cancer stages and MetS was also found (*X*^2^ = 7.97, *p* = 0.019). In addition, breast cancer survivors with MetS had significantly higher body weight (*p* = 0.032), waist circumference (*p* = 0.003), BMI (*p* = 0.023) and body fat percentage (*p* = 0.020). This study also examined the relationship between characteristics of breast cancer survivors in this study and their metabolic syndrome status as shown in Table 1 (Supplementary Material). The multiple logistics regression reports that sociodemographic and clinical characteristics were not related to metabolic syndrome status (*p* > 0.05).

## Discussion

MetS has been recognized as an important secondary target for the prevention of cardiovascular diseases and diabetes [31], as well as reducing the mortality rate among cancer survivors [32]. In this study, the Harmonized MetS definition that has been proposed in 2009 was used as a simple, useful and most updated guideline to diagnose MetS. Moreover, MetS prevalence was also reported by using WHO, ATP III and IDF diagnostic definitions for easier interpretation and comparison with other studies.

In this study, the prevalence of MetS among breast cancer survivors in East Coast of Peninsular Malaysia showed a higher percentage of subjects with MetS, up to half of the proportion of the investigated breast cancer survivors. When compared with the recent report by The Malaysian Breast Cancer Survivorship Cohort (MyBCC) study on the prevalence of MetS among newly-diagnosed breast cancer patients, higher proportion of breast cancer survivors with MetS was reported in the current study (48.4%) compared to 37.8% in MyBCC study according to IDF 2005 definition [6]. This difference can be attributed to the variation in breast cancer survival duration and ethnic composition percentage among the breast cancer survivors between these two studies. Furthermore, MetS prevalence among breast cancer survivors as reported in the current study was also similar, or higher than the data reported in other countries such as India – NCEP ATP III definition: 40.0% vs 40.0% [8], China – Harmonized definition: 50.5% vs 32.6% [9], Korea – Harmonized definition: 50.5% vs 43.9% [10], USA – Harmonized definition: 50.5% vs 26.1% [11], Denmark – NCEP ATP III definition: 40.0% vs 15.1% [13] and Brazil – Harmonized definition: 50.5% vs 48.1% [12] respectively.

The higher proportion of breast cancer survivors with MetS in Asian countries as compared to Western countries reflected that MetS has become more prevalent in developing countries when compared to its Western counterparts due to increasing economic development in lower to middle-income countries [33, 34]. This transition is also closely linked to unhealthy lifestyle changes associated with modernization such as increased sedentary behaviour [35], changes in dietary practices [36] and mental health deterioration [37]. As a result of increased mechanization and automation in daily activities in rural areas, there is also a rise in MetS prevalence in rural communities of the Asia-Pacific [34].

Contrarily, MetS prevalence among general women population had also been reported in numerous studies. In Malaysia, MetS prevalence among general Malaysian women in three nationwide studies were reported to range between 30.1–43.7% [15–17]. Besides, MetS prevalence among specific populations have also been reported, including among Kelantanese women (IDF definition: 32.2–36.6%) [38, 39], aborigines ‘Orang Asli’ women (Harmonized definition: 23.8%) [40], women in urban and rural areas (IDF definition: 10.8–39.3%) [41, 42], female university staff (NCEP ATP III definition: 21.4–45.3%) [43–45] and female government workers (Harmonized definition: 46.3%) [46]. Comparatively, higher prevalence of MetS was observed among the breast cancer survivors than the general women population, which supported previous reports describing the tendency of breast cancer survivors to be diagnosed with MetS [4, 5]. Since there is large gap between prevalence of breast cancer survivors and national prevalence, this strengthen the theory that MetS in breast cancer survivor might not be related to age but due to pre-existing cardiometabolic risk factors and comorbidities at any point of their lives. However, evidence whether the cancer itself attenuates the risk of MetS is still scarce. On the other hand, a recent meta-analysis has shown that MetS may predict the risk of cancer recurrence and mortality in women with breast cancer, particularly in Caucasians [47].

In the present study, among those with MetS as according to the Harmonized MetS definition (≥ 3 criteria), more than half of them met three MetS components, whereas 31.3 and 16.6% met four and five components respectively. However, when compared among all breast cancer survivors included in this study, the percentage of women meeting two MetS components (25.3%) was almost similar to those meeting three MetS components (26.3%). Furthermore, studies conducted among adults in China [48], Thailand [20], Netherland [23] and Nepal [49] also reported an almost similar, or even higher percentage of adults with two MetS components. If left with no intervention, this group of breast cancer survivors that was just below the borderline of MetS diagnosis would have a higher tendency to have a worse health condition or even being diagnosed with MetS in the future. Particularly, breast cancer survivors have been reported to have higher weight after a cancer diagnosis as compared to a year before being diagnosed with breast cancer [50, 51].

Moreover, the most prevalent abnormal MetS parameters among all breast cancer survivors were abdominal obesity, followed by hyperglycemia and hypertension. Previous studies have also reported an almost similar trend of the top three most prevalent abnormal MetS parameters [48, 52–54]. As increased waist circumference has been reported to be closely related with excess adiposity, impaired insulin sensitivity and other cardiometabolic factors, incremental changes in waist circumference would have detrimental effects to other MetS components [55, 56]. Moreover, increased blood pressure was also associated with central body fat distribution, independent of BMI and insulin resistance [57]. Meanwhile, dyslipidemia and hyperglycemia were more prevalent among breast cancer survivors with MetS. Therefore, targeting these conditions in the clinical settings should be the utmost priority in the effort to reduce MetS-related morbidity and mortality among breast cancer survivors in East Coast of Peninsular Malaysia.

Meanwhile, previous literatures have described the links between MetS and other sociodemographic and lifestyle factors among Malaysian adults, such as higher age, unemployment, working in shifts, postmenopausal status, living in urban area, lower socioeconomic status, Indian ethnicity, Chinese ethnicity and lower education level [6, 14–17, 38, 41, 58]. Specifically, these factors can be linked with other modifiable risk factors of MetS such as physical inactivity and unhealthy diets. According to Malaysian National Health and Morbidity Survey (NHMS) 2015, lower prevalence of physical activity was observed among older adults, Chinese ethnicity, those living in urban areas, having no formal education, retiree and lower household income [59]. Additionally, other studies have also reported physical inactivity among Indian ethnicity [16]. The NHMS 2015 survey also reported less intake of fruits and vegetables among Malays, those living in urban areas, having no formal education and middle-income group [59].

Similar to the findings of previous research, this study reported significant links between MetS and increased body weight [58, 60, 61], waist circumference [61–63], body fat percentage [61, 62] and BMI [9], except for total cholesterol level or LDL-c level. However, the findings revealed that MetS status is independent of sociodemographic and clinical characteristics. Older age, being Chinese ethnicity, being married, having low education level or being a housewife or pensioner is not a contributing factor for being at risk for MetS. Similarly, having a positive family history, having later or advanced cancer stage or longer duration of survivorship does not determine the risk of MetS. All other estrogen hormone related factors such as breastfeeding practices, being postmenopausal, oral contraceptive and hormone replacement therapy usage were not a significant risk factor for MetS as well among breast cancer survivors.

The differences in our findings may be attributed to several limitations of the study which should be addressed properly. Firstly, the breast cancer survivors included in this study were recruited only from Terengganu and Kelantan, hence the findings of this study might not represent all breast cancer survivors in Malaysia. Additionally, due to the sociodemographic characteristic and racial distribution of breast cancer survivors in Terengganu and Kelantan, data on breast cancer survivors from other ethnicities were very scarce, hence analysis on ethnicities and MetS in this study was very limited. It is also important to note the possibility that breast cancer survivors that agree to participate in this research might have more health-awareness compared to non-participants. Similarly, other important factors such as breast cancer subtype, physical activity and dietary intake were not reported in this study. Therefore, the links and their confounding effects on MetS could not be determined.

## Conclusion

To the best of our knowledge, this is the first study to report the prevalence of MetS among breast cancer survivors in East Coast of Peninsular Malaysia. MetS prevalence among breast cancer survivors in East Coast of Peninsular Malaysia was higher than normal population and in need of urgent attention. Therefore, in clinical settings, it is recommended to give utmost priority in improving triglycerides, blood glucose and HDL-c level of the breast cancer survivors in Malaysia to control MetS.

## Supplementary Information

**Additional file 1: Supplementary Material Table 1.** Relationship between characteristics of breast cancer survivors and metabolic syndrome.

## Data Availability

The datasets generated during and/or analyzed during the current study are available from the corresponding author on reasonable request.

## Abbreviations

BMI: Body Mass Index
BP: Blood Pressure
FBG: Fasting Blood Glucose
HDL-c: High Density Lipoprotein-cholesterol
IDF: International Diabetes Federation
IQR: Inter-Quartile Range
LDL-c: Low Density Lipoprotein-cholesterol
MetS: Metabolic Syndrome
MYR: Malaysian Ringgit
NCEP ATP III: National Cholesterol Education Program Adult Treatment Panel
NHMS: National Health and Morbidity Survey
SD: Standard Deviation
TG: Triglycerides
USD: United States Dollar
WC: Waist Circumference
WHO: World Health Organization
