# Supplementary material for: Prevalence of metabolic syndrome among breast cancer survivors in East Coast of Peninsular Malaysia
Source: BMC Public Health. 2021 Jan 28;21:238. doi: 10.1186/s12889-021-10288-9 (PMC7844947; doi:10.1186/s12889-021-10288-9)
Supplement: Supplementary file 1 — Additional file 1: Supplementary Material Table 1. Relationship between characteristics of breast cancer survivors and metabolic syndrome. [file 12889_2021_10288_MOESM1_ESM.docx]

**Supplementary Material**

Table 3. Relationship between characteristics of breast cancer survivors and metabolic syndrome

| Characteristics | OR | 95% CI | *p* |
| --- | --- | --- | --- |
| Age more than 50 years | 2.31 | 0.81 – 6.54 | 0.115 |
| Chinese ethnicity | 0.56 | 0.10 – 3.18 | 0.520 |
| Married | 0.95 | 0.30 – 3.00 | 0.938 |
| Low education level | 2.29 | 0.75 – 6.99 | 0.145 |
| Housewive or pensioner | 0.46 | 0.17 – 1.23 | 0.124 |
| Postmenopausal | 0.98 | 0.22 – 4.23 | 0.981 |
| Never breastfed | 0.44 | 0.09 – 2.09 | 0.305 |
| Use oral contraceptive pills | 0.55 | 0.22 – 1.41 | 0.218 |
| Use hormone replacement therapy | 0.48 | 0.14 – 1.61 | 0.237 |
| Positive family history | 1.17 | 0.42 – 3.25 | 0.754 |
| Later cancer stage | 1.22 | 0.34 – 4.39 | 0.759 |
| Cancer duration of more than 5 years | 0.77 | 0.29 – 2.01 | 0.597 |

Multiple logistics regression (Method=Enter). Model assumptions are fulfilled.

Coefficient of determination (R^2^) = 0.141

OR, Odds ratio; CI, Confidence interval.
